# Contribution of *Bacillus* Isolates to the Flavor Profiles of Vanilla Beans Assessed through Aroma Analysis and Chemometrics

**DOI:** 10.3390/molecules201018422

**Published:** 2015-10-09

**Authors:** Fenglin Gu, Yonggan Chen, Yiming Fang, Guiping Wu, Lehe Tan

**Affiliations:** 1 Spice and Beverage Research Institute, CATAS, Wanning 571533, Hainan, China; E-Mails: yonggan2006@163.com (Y.C.); fym870902@163.com (Y.F.); guiping81@163.com (G.W.); tlh3687@163.com (L.T.); 2 College of Bioscience and Technology, Qiongzhou University, Sanya 572022, Hainan, China; 3 College of Food Science and Technology, Huazhong Agricultural University, Wuhan 430070, Hubei, China

**Keywords:** vanilla, flavor profile, *Bacillus*, sensory analysis

## Abstract

Colonizing *Bacillus* in vanilla (*Vanilla planifolia* Andrews) beans is involved in glucovanillin hydrolysis and vanillin formation during conventional curing. The flavor profiles of vanilla beans under *Bacillus*-assisted curing were analyzed through gas chromatography-mass spectrometry, electronic nose, and quantitative sensory analysis. The flavor profiles were analytically compared among the vanilla beans under *Bacillus*-assisted curing, conventional curing, and non-microorganism-assisted curing. Vanilla beans added with *Bacillus vanillea* XY18 and *Bacillus subtilis* XY20 contained higher vanillin (3.58% ± 0.05% and 3.48% ± 0.10%, respectively) than vanilla beans that underwent non-microorganism-assisted curing and conventional curing (3.09% ± 0.14% and 3.21% ± 0.15%, respectively). Forty-two volatiles were identified from endogenous vanilla metabolism. Five other compounds were identified from exogenous *Bacillus* metabolism. Electronic nose data confirmed that vanilla flavors produced through the different curing processes were easily distinguished. Quantitative sensory analysis confirmed that *Bacillus*-assisted curing increased vanillin production without generating any unpleasant sensory attribute. Partial least squares regression further provided a correlation model of different measurements. Overall, we comparatively analyzed the flavor profiles of vanilla beans under *Bacillus*-assisted curing, indirectly demonstrated the mechanism of vanilla flavor formation by microbes.

## 1. Introduction

Vanilla (*Vanilla planifolia* Andrews) is a climbing plant native to Mexico [1]. The characteristic vanilla flavor (VF) is formed through curing, which produces the main aromatic constituent vanillin and over 200 other volatile compounds with delicate sweet fragrances [2]. Green vanilla beans contain glucosyl precursors of volatile compounds, the most important of which is glucovanillin. The glucosyl compound does not possess any interesting olfactory qualities until aglycone is released during the hydrolysis of *O*-glycosyl linkage [3].

Curing releases aglycone and, thus, liberates volatile compounds [4]. This process generally comprises four steps, namely, killing, sweating, drying, and conditioning. The entire curing process normally takes more than six months [5]. In conventional curing, mature green beans are first immersed in hot water for 3–5 min before subjecting to periodic sweating and drying. In the remaining part of the process, vanilla beans are allowed to sweat on wooden racks in a well-ventilated room and stored in small bundles of plastic vacuum bags at room temperature [6,7]. 

Vanillin content varies depending on the curing process of vanilla. Madagascar produces the best quality of vanilla (vanillin content of 2.0%–3.4%) from dry cured beans, whereas Indian vanilla contains 1%–2% vanillin [8,9]. Despite the co-existence of flavor substrates and relevant enzymes in vanilla beans, the enzymatic transformation of glucovanillin is not highly efficient [10]. Treating vanilla beans with exogenous pectinase, cellulose, β-d-glucosidase, and enzyme extract increases vanillin content [11,12,13]. Sreedhar *et al.* [7] combined acetone-dried red beet elicitor with pretreatments to accelerate curing of vanilla beans. They found that vanillin is formed within 10 days and that the vanillin content of beans with an elicitor is 1.7-fold higher than that of control beans and 3.23-fold higher than that of beans under conventional curing. However, the application of enzymes or biotic elicitors in pure form is impractical for the large-scale production of vanilla; hence, a cheap and simple method of increasing vanillin during vanilla curing should be developed. 

We have recently found that colonizing *Bacillus* in vanilla beans is involved in glucovanillin hydrolysis and vanillin formation during conventional curing [14]. Therefore, we speculated that *Bacillus* isolates can be used to develop a new, cheap, and simple method to increase vanillin production. However, the effects of *Bacillus*-assisted curing on the flavor profiles of vanilla remain unclear. 

This study investigated the flavor profiles of vanilla beans under *Bacillus*-assisted curing. Green vanilla beans were cured through conventional hot air processing and then sprayed with β-d-glucosidase-producing *Bacillus* isolates after drying. These strains assisted vanilla curing during the conditioning period. GC-MS and sensory investigations are commonly used to identify the volatile constituents of different samples [15,16]. Therefore, the volatile compounds released from the vanilla flavors (VFs) were analyzed through GC-MS, and the effects of *Bacillus*-assisted curing on the odor-active compounds were investigated. Electronic nose (E-nose) and sensory analysis were used to discriminate the VFs. A correlation analysis among the odor-active compounds, E-nose response, and sensory attributes were conducted to simultaneously interpret sample properties and variable relationships. Overall, this study provided insights into the contribution of *Bacillus*-assisted curing to vanilla flavor.

## 2. Results and Discussion

### 2.1. Effect of Bacillus Strains on Vanillin Formation 

**Figure 1:**
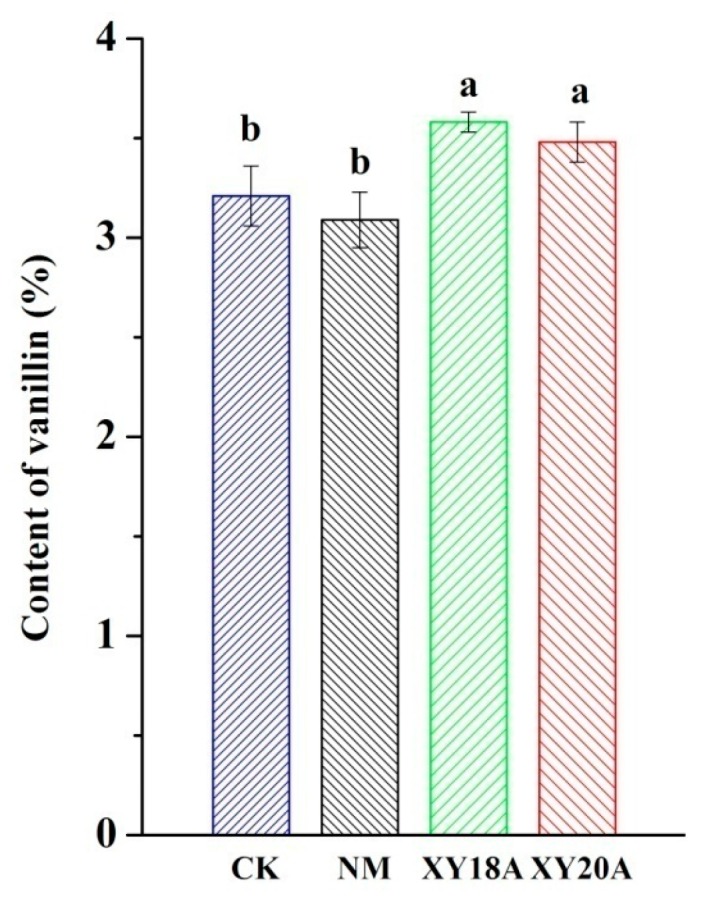
Vanillin content of vanilla beans under different curing processes. Data were subjected to Duncan’s test (*p* < 0.05). “a” and “b” mean that the significant differences of data subjected to Duncan’s test.

The HPLC profile of vanillin (percentage of dry weight) is shown in Figure 1. The obtained data were subjected to Duncan’s test (*p* < 0.05). The two samples added with *Bacillus* isolates significantly differed from the control sample (CK) and the non-microorganism-assisted curing sample (NM). A previous study suggested that the conversion of glucovanillin to vanillin during traditional curing in Reunion approaches only 40% of the hydrolytic capacity of β-d-glucosidase [17]. Curing under traditional field conditions yields vanillin between 1.5% and 3% on a dry weight basis, and thus, this approach may not exploit the full potential of β-d-glucosidase. Moreover, insufficient enzymatic action causes the incomplete hydrolytic release of vanillin during conventional curing [18]. Therefore, many studies focused on adding glycosyl hydrolases to increase vanillin production. Perera and Owen [19] reported that disrupting tissues through freezing-thawing and adding a mixture of hydrolyzing enzymes, such as cellulase, pectinase, and β-d-glucosidase, can liberate 7.00% ± 0.18% of vanillin from green vanilla beans. Vanilla beans mixed with a tea leaf enzyme extract in a suitable proportion can release 4.2% vanillin. The enzymes can be directly used to promote catalysis. However, the applications of these techniques in industrial curing practices are limited by the high cost of enzymes. In the present study, the vanillin contents of samples added with *Bacillus* isolates were significantly higher than those of CK and NM. This result suggests that the β-d-glucosidase-producing *Bacillus* isolates that colonize vanilla beans participate in the formation of vanillin and act as catalysis promoters to increase vanillin yield. Moreover, vanillin production may be significantly increased by optimizing curing, and this new, cheap method may be applied in industrial curing. 

### 2.2. Volatiles Identified in the Headspace of VFs

The main volatile compounds detected in the headspace of VFs are shown in Table 1. Forty-seven volatile compounds were extracted, including six acids, six alcohols, ten aldehydes, two bases, three esters, two ethers, three furans, three hydrocarbons, four ketones, one lactone, and seven phenols. These compounds were subdivided into three groups on the basis of their possible formation pathways: compounds formed from endogenous vanilla metabolism, compounds formed from exogenous *Bacillus* metabolism, and compounds formed from the interaction of *Bacillus* and vanilla metabolism. 

#### 2.2.1. Volatiles Formed from Endogenous Vanilla Metabolism

Forty-two compounds were found in NM (Table 1). Acids, alcohols, aldehydes, and phenolic compounds were the major volatiles. Aromatic compounds, such as homovanillic acid, 2-hormyl-1*H*-pyrrole, methyl 3-phenylacrylate, 2-(2-ethoxyethoxy)ethanol, butyl carbitol, 6-methyl-5-hepten-2-one, and *cis*-anethole were identified in the vanilla beans. These compounds have not been reported in previous studies [20,21]. Possibly, these compounds could be the markers of vanilla collected in Hainan, China. 

Sostaric *et al.* [22] developed a qualitative method to analyze the flavor volatiles present in vanilla extracts/flavorings through solid-phase microextraction (SPME). They proposed that different types of vanilla beans can be distinguished by marker compounds. Considerable amounts of *p*-methoxybenzoic acid methyl ester and 4-methoxybenzaldehyde were detected in the Tahitian extract, whereas only trace amounts of these compounds were found in the Indonesian and Bourbon extracts. The Indonesian and Bourbon extracts were distinguished by the different relative amounts of key components, such as hexanoic acid, 5-propenyl-1,3-benzodioxole, and ethyl nonanoate. By contrast, *p*-methoxybenzoic acid methyl ester, 5-propenyl-1,3-benzodioxole, and ethyl nonanoate were not detected in the present study. This result reflects that vanilla beans collected from different areas can be distinguished by the marker compounds. 

**Table 1:** Volatile compounds detected in the headspace from cured vanilla beans obtained using various curing process.

Compounds	RI_DB-WAX_	RI_literature_	CK	NM	XY18A	XY20A
*Acids*						
Acetic acid	1432	1446	*	*	*	*
Propionic acid	1524	1530	*	*	*	*
Butanoic acid	1616	1621	*	*	*	*
Hexanoic acid	1837	1840	*	–	*	*
Homovanillic acid	2312	–	–	*	*	*
Benzoic acid	2493	2415	–	–	*	–
*Alcohols*						
1-Octanol	1548	1555	*	*	*	*
2,3-Butanediol	1566	1566	*	*	*	*
Benzyl alcohol	1851	1869	*	*	*	*
Phenylethyl alcohol	1883	1906	*	*	*	*
*p*-Anisyl alcohol	2248	2191	*	*	*	*
Vanillyl alcohol	2769	–	*	*	*	*
*Aldehydes*						
Hexanal	1048	1080	*	*	*	*
Octanal	1273	1289	*	*	*	*
Heptenal	1304	1328	*	*	*	*
Nonanal	1377	1392	*	*	*	*
Benzaldehyde	1493	1519	*	*	*	*
4-Methoxybenzaldehyde	1982	2018	*	*	*	*
Piperonal	2184	–	*	*	*	*
Vanillin	2530	2578	*	*	*	*
Isovanillin	2570	–	*	*	*	*
*p*-Formylphenol	2914	–	*	*	*	*
*Bases*						
2-Acetyl-1*H*-pyrrole	1940	1991	*	–	*	*
2-Formyl-1*H*-pyrrole	1988	1965	*	*	*	*
*Esters*						
2-Phenylethyl acetate	1786	1822	*	–	*	*
Methyl 3-phenylacrylate	2040	2035	*	*	*	*
4-Formyl-2-methoxyphenyl acetate	2467	–	*	*	*	*
*Ethers*						
2-(2-ethoxyethoxy)ethanol	1603	1577	*	*	*	*
Butyl carbitol	1776	1793	*	*	*	*
*Furans*						
Furfural	1439	1452	*	*	*	*
2-Acetylfuran	1479	1494	*	*	*	*
5-Methylfurfural	1544	1568	*	*	*	*
*Hydrocarbons*						
3-Carene	1125	1146	*	*	*	*
Limonene	1177	1198	*	*	*	*
Tetradecane	1392	1400	*	*	*	*
*Ketones*						
Acetoin	1263	1283	*	*	*	*
6-Methyl-5-hepten-2-one	1320	1342	*	*	*	*
6,10,14-Trimethyl-2-pentadecanone	2110	2115	*	*	*	*
Apocynin	2600	–	–	–	*	–
*Lactone*						
Butyrolactone	1587	1643	*	*	*	*
*Phenols*						
*cis*-Anethole	1794	1845	*	*	*	*
Guaiacol	1831	1857	*	*	*	*
Creosol	1926	1927	*	*	*	*
Phenol	1979	2003	*	*	*	–
4-Methylphenol	2056	2074	*	*	*	*
2-Methoxy-4-vinylphenol	2164	2192	*	*	*	*
4-(Ethoxymethyl)phenol	2514	–	–	*	*	*

Literature RI obtained from [23,24]; * detected; – not detected, also in RI_literature_ refers to not found.

#### 2.2.2. Volatiles Formed from Exogenous *Bacillus* Metabolism

Five compounds (hexanoic acid, benzoic acid, 2-acetyl-1*H*-pyrrole, 2-phenylethyl acetate, and apocynin) were solely identified in the VFs of vanilla beans under *Bacillus*-assisted curing (Table 1). These compounds may indicate *Bacillus* strains that colonize the vanilla beans. Studies confirmed that microbial activities involved in curing can directly provide aromatic compounds [25,26]. However, microbial contribution to the overall vanilla flavor has only been suggested and not fully investigated. 

Hexanoic acid was detected in CK, XY18-assisted curing sample (XY18A), and XY20-assisted curing sample (XY20A) but not in NM, revealing that this acid originated from microbial metabolism. Hartman [27] reported that hexanoic acid is unique to Mexican vanilla. Other researchers used SPME coupled with GC-MS to profile vanilla extracts. They revealed that this method can distinguish Bourbon and Indonesian vanilla extracts on the basis of the quantity of hexanoic acid. Thus, they proposed the use of this method to identify adulterated products and determine vanilla extract or flavoring types [22]. Another study revealed that Mexican and Ugandan vanilla beans contain 0.05 and 0.38 mg/kg hexanoic acid, respectively [21]. Differences were found in these studies, possibly indicating that hexanoicacid is not the marker compound of vanilla species. Perhaps, the existence of hexanoic acid was related to the microbe. 

#### 2.2.3. Volatiles Formed from Interaction of *Bacillus* and Vanilla Metabolism

The overall flavor of *V.planifolia* is contributed by 26 odor-active compounds, including guaiacol, valeric acid, 2,3-butanediol, and 2-heptenal, whose concentrations are a thousand times lower than that of vanillin and whose intensities are similar to that of vanillin [28]. Nine odor-active compounds were selected to quantify and characterize the interaction of *Bacillus* and vanilla metabolism (Table 2). In the present study, the percentage of butanoic acid in XY20A (0.060% ± 0.0226%) was significantly higher than that in NM (0.024% ± 0.0185%). This result indicates that strain XY20 can increase the production of butanoic acid from vanilla metabolism. Butanoic acid has been extensively used in the food industry as a flavor additive that increases buttery-like fragrance [29]. More than 10 butyrate-producing anaerobic bacterial species belonging to the genera *Clostridium*, *Butyrvibrio*, *Butyribacterium*, *Eubacterium*, *Fusobacterium*, *Megasphera*, and *Sarcina* have been investigated for their potential applications [30,31]. The results of the present study found that aerobic *Bacillus* isolates can increase butanoic acid production, revealing a different metabolic pathway potential for butanoic acid production and regulation. 

XT20A had significantly lower acetoin content than CK, NM, and XT18A (Table 2). This result indicates that strain XY20 degraded acetoin during curing. Similarly, acetoin is used by *B. subtilis* when other carbon sources are depleted. The catabolism is induced by acetoin and repressed by glucose in the growth medium [32]. Therefore, additional strains not only increase the target compounds but also degrade the non-target flavor compounds. 

**Table 2:** Aroma compounds found in SPME extracts of four VF samples.

No.	Compounds	Quantities(%) *			
		CK	NM	XY18A	XY20A
A1	Acetic acid	0.080 ± 0.0081 ^a^	0.080 ± 0.0121 ^a^	0.087 ± 0.0076 ^a^	0.086 ± 0.0078 ^a^
A2	Butanoic acid	0.026 ± 0.0154 ^a^	0.024 ± 0.0185 ^a^	0.033 ± 0.0042 ^ab^	0.060 ± 0.0226 ^b^
A3	2,3-Butanediol	0.021 ± 0.0019 ^a^	0.023 ± 0.0009 ^a^	0.023 ± 0.0007 ^a^	0.024 ± 0.0026 ^a^
A4	Vanillyl alcohol	0.004 ± 0.0024 ^a^	0.002 ± 0.0005 ^a^	0.003 ± 0.0002 ^a^	0.003 ± 0.0033 ^a^
A5	Heptenal	0.010 ± 0.0098 ^a^	0.011 ± 0.0110 ^a^	0.009 ± 0.0018 ^a^	0.011 ± 0.0018 ^a^
A6	Acetoin	0.033 ± 0.0058 ^b^	0.026 ± 0.0038 ^ab^	0.030 ± 0.0011 ^b^	0.021 ± 0.0051 ^a^
A7	Guaiacol	0.028 ± 0.0013 ^b^	0.022 ± 0.0035 ^a^	0.023 ± 0.0013 ^a^	0.025 ± 0.0028 ^ab^
A8	Creosol	0.004 ± 0.0012 ^a^	0.004 ± 0.0007 ^a^	0.004 ± 0.0001 ^a^	0.004 ± 0.0004 ^a^
A9	2-Methoxy-4-vinylphenol	0.003 ± 0.0017 ^a^	0.003 ± 0.0011 ^a^	0.003 ± 0.0003 ^a^	0.005 ± 0.0016 ^a^

* Quantities (mean ± standard deviation, average of triplicate) for each component within a row with different letters are significantly different according to Duncan’s test (*p* < 0.05).

### 2.3. E-nose Analysis of VFs

**Figure 2:**
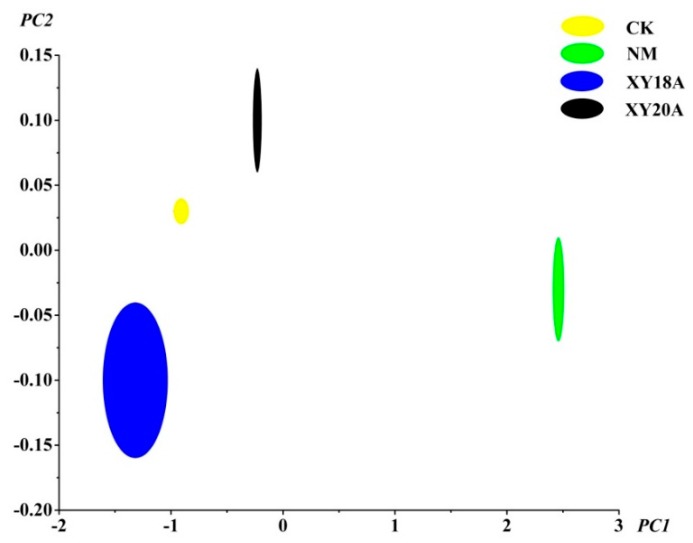
Plot of the first two principal components based on E-nose data.

E-nose data were analyzed through principal component analysis (PCA) to determine the discrimination of measurements. The PCA pattern of E-nose data for VFs is shown in Figure 2. Principal component 1 (PC1) accounted for the major differences (99.676%) in variances, whereas principal component 2 (PC2) accounted for the minor differences (0.3039%). A score plot for PC1 and PC2 indicated a certain systematic variation in the data with regard to the different curing processes. As shown in Figure 2, the samples under microorganism-assisted curing were located in the left half of the plot, whereas with the samples that did not undergo non-microorganism-assisted curing were located in the right half. This result indicates the possible correlation of PC1 with microorganism assistance. CK, XY18A, and XY20A cured under the assistance of different microorganisms were separated in PC2. CK was located in the middle of XY18A and XY20A. The flavor profile of the vanilla beans that underwent *Bacillus*-assisted curing was associated with PC2. These results indicate that E-nose is a potentially feasible method for the rapid identification of VFs with different curing processes [13,32].

### 2.4. Sensory Characteristics of VFs

**Figure 3:**
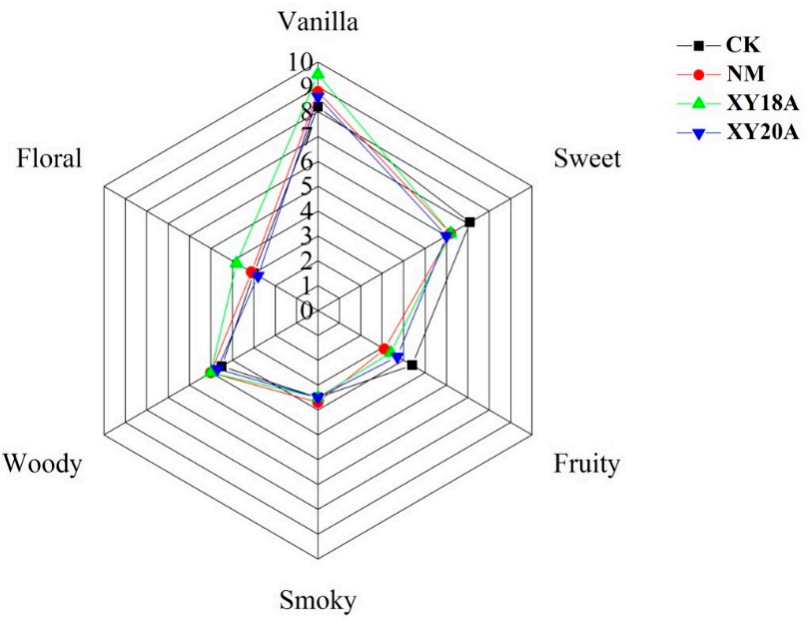
Sensory profile of vanilla beans from the different curing processes. Data were subjected to Duncan’s test (*p* < 0.05).

The flavor profiles of vanilla beans are shown in Figure 3. The data on all six sensory attributes (*i.e.*, vanilla, sweet, fruity, smoky, woody, and floral) of the four VFs were subjected to radar analysis. ANOVA indicated no significant differences (*p* < 0.05) in the intensity of smoky and woody attributes among the samples. Previous studies reported that samples that undergo chemical enzyme-assisted curing produce an intense unpleasant odor in woody and beany notes [11,33]. In the present study, *Bacillus*-assisted curing increased vanillin production without generating any unpleasant sensory attribute. Therefore, external treatment with *Bacillus* isolates can be used for the commercial production of high-quality vanilla.

### 2.5. Comparison of the Characteristic Compounds, E-nose Response, and Sensory Attributes of VFs

**Figure 4:**
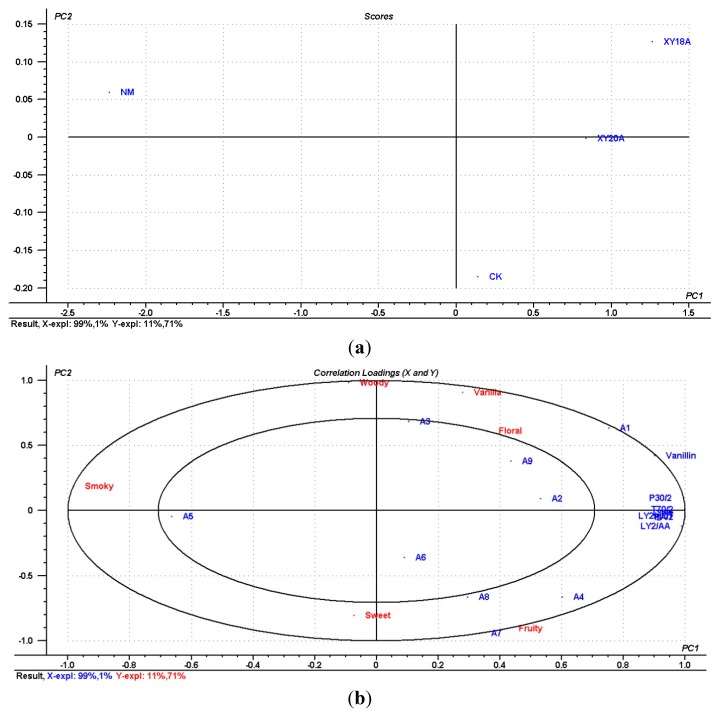
Correlation of the characteristic compounds, E-nose response, and sensory attributes of VFs. (**a**) PLSR correlation loading plot of selected odor-active compounds, E-nose response, and sensory attributes for four VFs. Selected compounds A1–A9 correspond to the coded compounds in Table 2. (**b**) Plot of the first two principal components based onselected odor-active compounds, E-nose response, and sensory attributes for four VFs.

Partial least squares regression (PLSR) was performed on four samples to compare the different data from the odor-active compounds, E-nose measurement, and sensory evaluation. The derived PLSR model included two significant PCs explaining 99% of the cross validated variance (Figure 4a). The outer ellipse indicated 100% of the explained variance, whereas the inner ellipse indicated 50% of the explained variance. Thus, the sensory parameters between the two ellipses might be correlated with the odor-active compounds and E-nose response, whereas those inside the inner ellipse poorly correlated. Vanilla and floral attributes significantly correlated with vanillin. The resultant correlation loading plot of the first two components (Figure 4a) indicates that six gas sensors are classified into one group and that the sensors show similar responses to the VFs. Vanillin was well described by the six gas sensors. The results agree with our previous finding that these gas sensors are sensitive to vanillin associated with vanilla attributes [34].

The combined data were analyzed through PCA to discriminate the four samples. The PCA pattern is shown in Figure 4b. XY18, XY20, and CK shared similar attributes, compared with NM, and XY18 and XY20 correlated with each other and had significant vanilla and floral attributes. NM had fewer odors and poorly correlated with the three other samples but had a significant smoky attribute (Figure 4a,b). The results confirmed that the characteristic vanilla flavor produced through microorganism-assisted curing was stronger than that produced through non-microorganism-assisted curing.

## 3. Experimental Section 

### 3.1. Samples and Chemicals

*V. planifolia* was used in this study. Fruits were pendulous, narrow cylindrical capsules, obscurely three-angled, and longitudinally split when mature. They were collected in Hainan, China. All chemicals were analytical grade. Vanillin, methanol, alcohol, acetic acid, and NaOCl were purchased from Sinopharm Chemical Reagent Co. Ltd. (Shanghai, China).

### 3.2. Sample Preparation and Analysis 

β-d-Glucosidase-producing *B. vanillea* XY18 and *B. subtilis* XY20 were stored in our lab and selected for this study. The two isolates were cultured on Luria-Bertani medium for 24 h. The culture was centrifuged at 7000 rpm for 1 min and washed with sterile water before using for assistance curing. 

The test and control groups (traditional curing) each containing 100 g of vanilla beans were cured through hot air processing [34]. After eight weeks of curing, the control beans were size-reduced (3–5 cm) and conditioned in vacuum. The test beans were double-treated with 75% alcohol and NaOCl (1% available Cl) and then size-reduced. NM was directly conditioned in vacuum, whereas XY18A and XY20A were sprayed with strains before vacuum conditioning.

#### 3.2.1. Determination of Vanillin 

Vanillin was determined following the method described by Dong *et al.* [34]. The samples were ground into powder (0.5 g) and placed in 150 mL volumetric flasks. Vanilla bean powder was dissolved in a 70% (*v*/*v*) ethanol–water solution at a 0.5 g/50 mL sample/solvent ratio. Microwave irradiation (100 W, 20 min) was applied to the samples. The extracts were analyzed in triplicate using HPLC (Agilent 1260, Waldbronn, Germany). The samples were filtered through a 0.45 μm filter prior to analysis. HPLC equipped with a Zorbax Eclipse Plus C18 column (4.6 mm × 100 mm, 3.5 μm Agilent) was used to determine vanillin content. Isocratic elution at 1.0 mL/min flow rate was performed using a mixture of 20% methanol and 80% acidified water. Water (1000 mL) was acidified with 5 mL of acetic acid. The total injection volume for analysis was 5 μL. A variable wavelength detector (VWD) at 280 nm was used, and the column temperature was maintained at 26 °C. The compounds were quantified using the external standard technique. 

#### 3.2.2. GC-MS Analysis 

Vanilla beans (1 g) were ground into powder and analyzed by HS-SPME/GC-MS. The new CAR/PDMS fiber used in this study was 75 μm thick. Three replicates of a single treatment were made and the sequence of every replicate was sampled randomly. The samples were heated for 40 min at 80 °C, and the fiber was exposed to the headspace in the final 20 min. Desorption of fiber was 5 min at 250 °C. GC-MS analysis was performed using an Agilent 7890A gas chromatograph coupled to an Agilent 5975C quadrupole mass spectrometer. Volatiles were separated on a DB-WAX (J&W Scientific, Folsom, CA, USA) fused silica capillary column (30 m, 0.25 mm and 0.25 μm film thickness). The column temperature was set at 40 °C for 3 min, raised to 90 °C at 3 °C/min, to 120 °C at 2 °C/min, and then to 245 °C at 3 °C/min, which was held for 20 min. The injector was heated at 250 °C. The samples were injected in splitless mode. The electron impact energy was 70 eV, and the ion source and quadrupole temperatures were set at 230 °C and 150 °C, respectively. Electron impact mass spectra were recorded in the 40–600 amu range at 1 s intervals. Compounds were identified on the basis of the linear RI, interpretation of their mass spectra, and the data available in the spectral library (Wiley/NIST Libraries, Weinheim, Germany) of the instrument or comparison with available authentic compounds. The linear RI was calculated using n-alkanes (C8–C40) as a reference. Component concentrations were calculated on the basis of the ratio of the total deconvoluted area of each component against vanillin. The extraction of odor-active compounds from the VFs was performed in triplicate, and the concentration of these compounds was calculated using the ratio of peak area with vanillin.

#### 3.2.3. E-nose Analysis 

Samples were analyzed using a gas-sensor array technique (Alpha M.O.S., Toulouse, France) with six metal oxide sensors and a headspace auto-sampler. Homogenized samples (0.5 g) were weighed into 10 mL glass headspace vials (the samples were analyzed in triplicate). The vials were sealed, and the samples were equilibrated at 50 °C for 5 min. The injection volume was 1500 μL, the acquisition delay was 210 s, and the syringe temperature was 60 °C. The response data were analyzed using E-nose software (Alpha Soft version 3.0.0, Toulouse, France).

#### 3.2.4. Sensory Evaluation

Sensory evaluation was carried out in accordance with previously described methods with slight modifications [35,36,37]. The evaluation was performed in booth rooms maintained at a temperature of 22 ± 2 °C and a relative humidity of 45% ± 5% with fluorescent lights. Quantitative descriptive analysis involving a 15 cm line scale, wherein 1.25 cm was anchored as low and 13.75 cm as high, was conducted [38].

Fifteen panelists were trained over three sessions for quantitative sensory analysis. The staff were familiar with the sensory analysis techniques used in plantation products, flavor technology, and related fields. One extensive training session that lasted for 2 h was conducted to familiarize the assessors with the descriptors and intensity scales. The training included the development of a common lexicon of the sensory attributes in the evaluation. Dominant flavor notes of vanilla and appropriately diluted reference compounds that correspond to the flavor notes were provided to assist the panelists in selecting descriptors [39]. The reference compounds—vanillin, aldehyde, citral, smoke-like, wood-like, and floral-like—were used to assist vanilla, sweet, fruity, smoky, woody and floral odor description. Each sample was randomly served. The panelists were asked to indicate the perceived vertical line on the scale and write the code of the sample close to the line. Cured vanilla pods (0.5 g) were pulverized and placed in a 50 mL conical flask sealed with a stopper. The panelists sniffed the headspace generated in the flask and indicated the intensity of the perceived attribute on a scorecard. Between two successive evaluations, a time interval of 10 min was given for the buildup of aroma. The mean values of intensity ratings were calculated, and a schematic of the flavor profile was presented in the form of a spider-web diagram.

#### 3.2.5. Data Analysis

Data from the descriptive analysis were evaluated through ANOVA using SPSS 20. The correlations among odor-active compounds, E-nose response, and sensory attributes were analyzed through PLSR using Unscrambler version 9.7 (CAMO ASA, Oslo, Norway). PLSR was performed as previously described by Song *et al.* [40].

## 4. Conclusions

Forty-seven volatile compounds were identified from the VFs. Forty-two of these compounds were detected in NM. The vanilla beans under *Bacillus*-assisted vanilla curing and conventional curing produced more vanillin than those under non-microorganism-assisted curing. Thus, colonizing *Bacillus* in vanilla beans is involved in glucovanillin hydrolysis and vanillin formation during conventional curing. Moreover, selected β-d-glucosidase-producing *Bacillus* isolates can be used to increase vanillin production without generating any unpleasant sensory attribute. E-nose and the relative marker compounds can be used to discriminate the VFs of *Bacillus*-assisted curing.

## Abbreviations

VF	Vanilla flavor
CK	Control (hot air processing)
NM	Non-microorganism-assisted curing sample
XY18A	XY18-assisted curing sample
XY20A	XY20-assisted curing sample
GC-MS	Gas chromatography-mass spectrometry
SPME	Solid-phase microextraction
CAR/PDMS	Carboxen/polydimethylsiloxane
E-nose	Electronic nose
HPLC	High-performance liquid chromatography
PLSR	Partial least squares regression
PCA	Principal component analysis
RI	Retention index
